# Allotropes of tellurium from first-principles crystal structure prediction calculations under pressure

**DOI:** 10.1039/c8ra07843b

**Published:** 2018-11-27

**Authors:** Yuan Liu, Shunbo Hu, Riccarda Caputo, Kaitong Sun, Yongchang Li, Guodong Zhao, Wei Ren

**Affiliations:** Materials Genome Institute, International Centre for Quantum and Molecular Structures, and Department of Physics, Shanghai University Shanghai 200444 China renwei@shu.edu.cn; Shanghai Key Laboratory of High Temperature Superconductors, Shanghai University Shanghai 200444 China; International Centre for Quantum and Molecular Structures, Shanghai University Shanghai 200444 China

## Abstract

We investigated the allotropes of tellurium under hydrostatic pressure based on density functional theory calculations and crystal structure prediction methodology. Our calculated enthalpy-pressure and energy-volume curves unveil the transition sequence from the trigonal semiconducting phase, represented by the space group *P*3_1_21 in the range of 0–6 GPa, to the body centered cubic structure, space group *Im*3̄*m*, stable at 28 GPa. In between, the calculations suggest a monoclinic structure, represented by the space group *C*2/*m* and stable at 6 GPa, and the β-Po type structure, space group *R*3̄*m*, stable at 10 GPa. The face-centered structure is found at pressure as high as 200 GPa. As the pressure is increased, the transition from the semiconducting phase to metallic phases is observed.

## Abstract 2

Through first-principles simulations, we suggest the phase stability of the allotropic transition sequence of tellurium from the trigonal structure up to the cubic structure.

## Graphical abstract

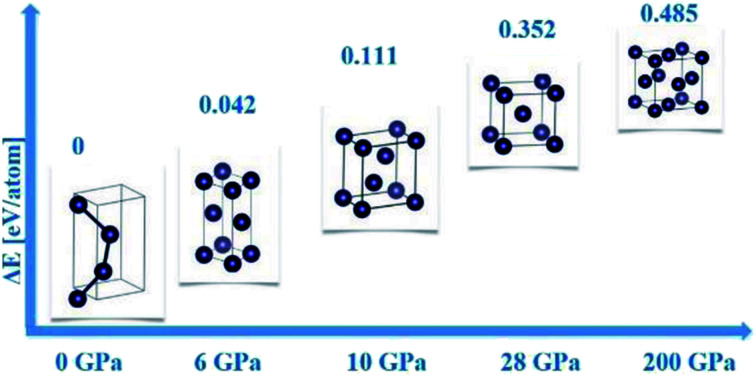

## Introduction

Tellurium is a semiconductor with a high thermoelectric figure of merit comparable to GeSi,^1^ BiSb^2^ or other well-known materials.^3–5^ The chiral tellurium has been investigated for its anisotropic lattice thermal conductivity and p-type transport properties from anisotropic hole pockets of the Fermi surface.^6,7^ In addition, very recently, structures with few-layers of tellurium were studied as two-dimensional topological materials.^8,9^ In fact, it presents topological properties for electronic transport, benefitting from the nonsymmorphic screw symmetry, the lone-pair electrons and the strong spin–orbit interaction.^10–12^ For the ground-state chiral tellurium, the band splitting and band inversion were found upon the application of shear strain or pressure^13–15^ together with the existence of topological Weyl nodes near the Fermi level.^13,14^

Regarding the crystal structure, tellurium undergoes several phase transitions under pressure. Though there is a lack of consensus of the exact phase transitions, the sequence reported in the literature suggests the transition from the ground state, represented in the trigonal space group *P*3_1_21 to a puckered layer phase at 4 GPa,^16^ and then to the monoclinic space group *P*2_1_ and to an orthorhombic structure in the pressure range of 6.6–10 GPa.^16,17^ Powder X-ray diffraction (XRD) measurements at 5.3 GPa and 11.5 GPa led to the conclusion that the structure at 11.5 GPa is of the β-Po type (space group *R*3̄*m*).^18^ Therefore, the overall phase transitions are from the trigonal, space group *P*3_1_21 (Te-I) to the monoclinic, space group *P*2_1_ (Te-II) at about 4 GPa;^16^ from Te-II to the orthorhombic phase (Te-III) at about 7 GPa; from Te-III to the rhombohedral phase in the trigonal space group *R*3̄*m* (Te-IV) at about 10.5 GPa; from Te-IV to the body-centered cubic phase (Te-) at 27 GPa.^18,19^ To the best of our knowledge, the symmetry representation of the orthorhombic phase is not reported in the literature. In contrast to that, the X-ray structural analysis^20^ ruled out the second-order phase transition from the monoclinic *P*2_1_ to the orthorhombic phase and then to the trigonal *R*3̄*m*, and suggested that the monoclinic should be represented instead in the *C*2/*m* space group.^20^ Therein the authors^20^ suggested the phase transitions as increasing the pressure as follows: trigonal → puckered monoclinic → monoclinic → rhombohedral → body-centered cubic (bcc). Hejny and McMahon^21,22^ further showed that Te-II was a triclinic structure and Te-III was an incommensurate monoclinic structure. Accordingly, the phase transition sequence they proposed is from the trigonal phase, consisting of helical spiral chains, to the commensurate triclinic structure, comprising puckered layers that contain zig-zag chains with alternating long and short bonds (such values are similar to Se-III *C*2/*m*),^21^ to the incommensurate monoclinic structure with a superspace group *I*′*2*/*m*(0q0)s0 (with *a* = 3.288 Å, *b* = 4.010 Å, *c* = 2.589 Å, *β* = 112.98°),^22^ and finally to a bcc phase. Therefore, the phase transition sequence investigated by Hejny *et al.* is as follows: trigonal → triclinic → monoclinic (→ β-Po rhombohedral under high pressure and high temperature) → bcc.^21,22^ In the last decade, *via* synchrotron radiation experiments, it was found that the cubic structure (bcc) transforms to a higher-pressure phase with face-centered cubic (fcc) superlattice structure at 100 GPa, which further transforms into the fcc phase at 255 GPa.^23^ Clearly, there are still controversies and ambiguities in the question of the crystal structures of tellurium under pressure from the experiments.^16,18–22^

Therefore, it is highly advisable a theoretical and systematic study of the crystal structures of Te by using the state-of-the-art methodologies in crystal structure prediction combined with total energy calculations.^24–27^ In the present work, we employed first-principles crystal structure calculations, by using the particle swarm optimization (PSO) method, as implemented in CALYPSO^28^ code to generate the structures at different pressures and the Vienna Ab initio Simulation Package (VASP)^29,30^ for the total energy optimizations. The structural stability was checked *via* phonon calculations.^31^ A comparison with the experimentally reported structures was possible *via* X-ray diffraction patterns calculations.

### Computational methodology

The crystal structure analysis based on the particle swarm optimization (as implemented in the CALYPSO package)^28^ was employed to search for possible low-energy structures of tellurium. The entire ensemble of structures were generated by using an initial cell containing 3, 4, 6 atoms and 12 atoms with 30 generations. Each generation contains 20 structures, 60% of these structures are generated from lowest-enthalpy structure provided by the previous generation, and evolved using particle swarm optimization, while the remaining 40% will be generated randomly. The pressure scan was run by steps of 2 GPa in the range of 0–32 GPa, and by steps of 50 GPa in the range of 100–250 GPa. The energy calculations and the geometry optimizations were conducted using VASP^29,30^ with the projector augmented wave (PAW) method.^32,33^ The reliability of the pseudopotential approach has also been confirmed by the full-potential linearized augmented plane waves approach. The generalized gradient approximation with the Perdew–Burke–Ernzerhof (PBE) functional for the exchange correlation was employed.^34^ A plane-wave basis with a cutoff energy of 550 eV was used to expand the wave functions. The Monkhorst–Pack *k*-points mesh was chosen to be no larger than 0.04 Å^−1^. Here, we also adopted the screened hybrid functional HSE06 (ref. 35 and 36) to calculate the band gap the *P*3_1_21 phase, using a fixed HF : GGA mixing ratio of 25 : 75 and a screening parameter of 0.2 Å^−1^. We checked the structure stability *via* phonon calculations, which are performed using 2 × 2 × 2 supercells, as implemented in the Phonopy package.^31^ X-ray powder diffraction (XRD) patterns were simulated by using the Reflex tool implemented in the Materials Studio (MS) package software. The diffraction patterns were calculated over a 2*θ*-range from 5° to 45° with an incremental step size of 0.05° and for the diffractometer, the silver (Kα = 0.5594075 Å) radiation source, in order to compared our results with the experimental data reported in the literature.^20^

## Results and discussion

**Fig. 1:**
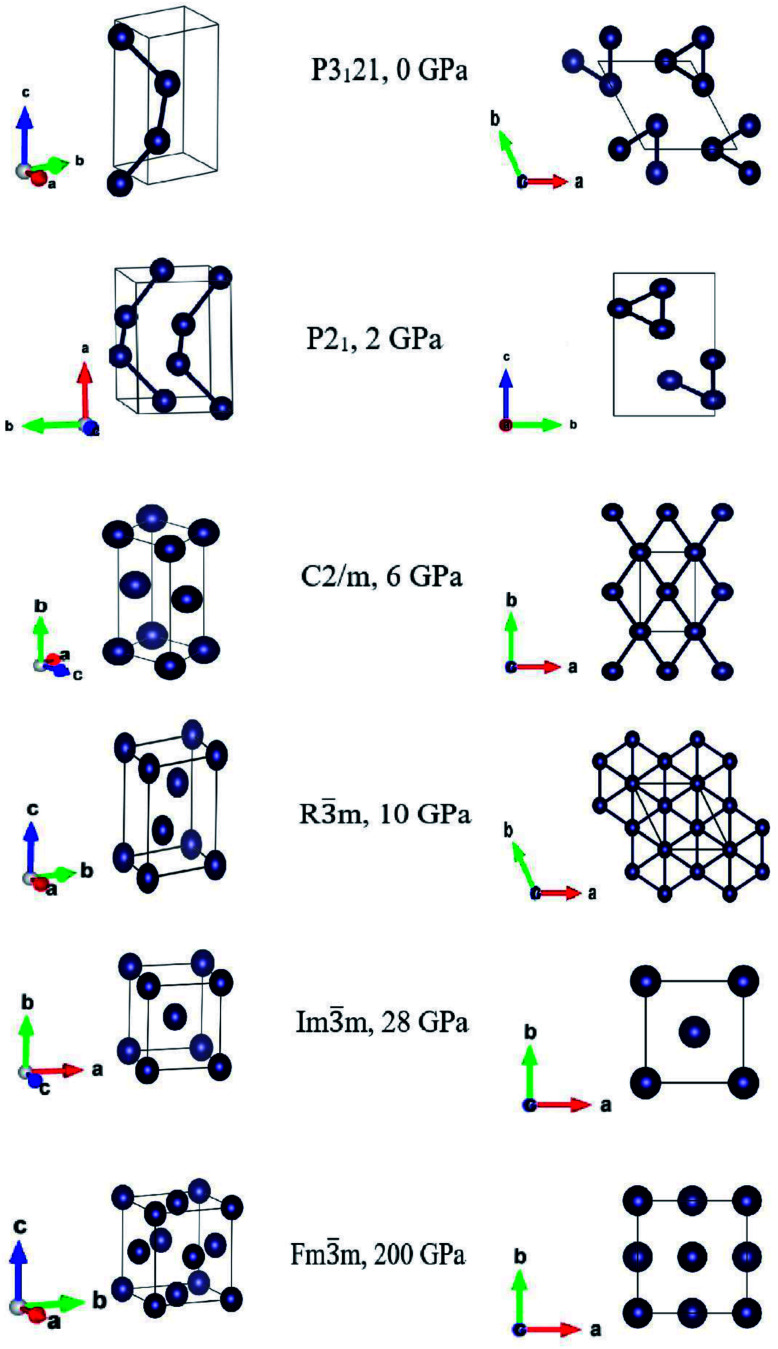
3D-views (left panels) and top-views (right panels) of the optimized structures at the corresponding pressures at which they exhibited lattice stability. For completeness, we show the monoclinic *P*2_1_, found at 2 GPa, which transformed back into the trigonal *P*3_1_21 after full geometry optimization.

Our first-principles crystal structure prediction results confirmed that the ground state is the trigonal *P*3_1_21 phase and that it is stable in the range of 0–6 GPa. In the range 6–10 GPa we found a monoclinic *C*2/*m* structure, which transformed into the trigonal *R*3̄*m* at pressure higher than 10 GPa. As increasing further the pressure this trigonal structure in the rhombohedral representation transformed into a body center cubic structure *Im*3̄*m*, stable at 28 GPa. At very high pressures, from 100 GPa the bcc structure transformed into the face centered cubic *Fm*3̄*m* phase, stable at 200 GPa. The three-dimensional and top views of the corresponding conventional unit cells are displayed in Fig. 1, and the structural and thermodynamic data are reported in Table 1.

**Table 1:** Structural and thermodynamic data. The values of the bulk modulus (*B*_0_) and its first derivative 
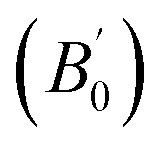
 are obtained by interpolation of the energy-volume curves (at *P* = 0) reported in Fig. 3 and by using the eqn (1). The lattice angle *β* of the monoclinic *C*2/*m* is 89.245°

Space group	Pressure (GPa)	*a* (Å)	*b* (Å)	*c* (Å)	*E*–*E* (trig) (eV)	*V*/atom (Å/atom)	*B*_0_ (GPa)	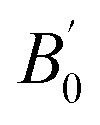	Site	*x*	*y*	*z*
*P*3_1_21^a^	0	4.514	4.514	5.955	0	34.833	50.883	4.664	3a	0.2691	0	1/3
*C*2/*m*^b^	6	8.138	4.701	3.692	0.042	32.782	51.863	4.306	2a	0	0	0
									4i	0.324	0	0.325
*R*3̄*m*	10	4.764	4.764	3.992	0.111	29.419	61.243	5.655	3a	0	0	0
*Im*3̄*m*	28	3.515	3.515	3.515	0.352	21.714	49.124	4.352	2a	1/2	1/2	1/2
*Fm*3̄*m*	200	3.819	3.819	3.819	0.485	27.664	51.863	4.306	4b	0	0	0

a The lattice parameters of the trigonal *P*3_1_21 phase from experiments^37^ are *a* = 4.527 Å, *c* = 5.929 Å

b The lattice parameters of the monoclinic *C*2/*m* phase from experiments^20^ are *a* = 8.4682(14) Å, *b* = 4.7424(8) Å, *c* = 3.9595(7) Å, *β* = 88.112(11)°. In the standard representation, *β* = 91.888° and the atoms are on the positions (2a) in (0, 0, 0) and (4i) in (0.324, 0, 0.325).

The *P*3_1_21 phase has a trigonal crystal structure with helical chains parallel to the crystallographic *c*-direction. As reported,^16,38^ we also obtained the monoclinic *P*2_1_ phase in our crystal structure calculations at 2 GPa, but it transformed back into the trigonal structure *P*3_1_21 after full geometry optimization. We find an excellent consistency between the crystal structures from our CALYPSO search^28^ and the previous experiments.^20^ The lattice parameters of the trigonal *P*3_1_21 structures are in good agreement with the experimental values.^37^

**Fig. 2:**
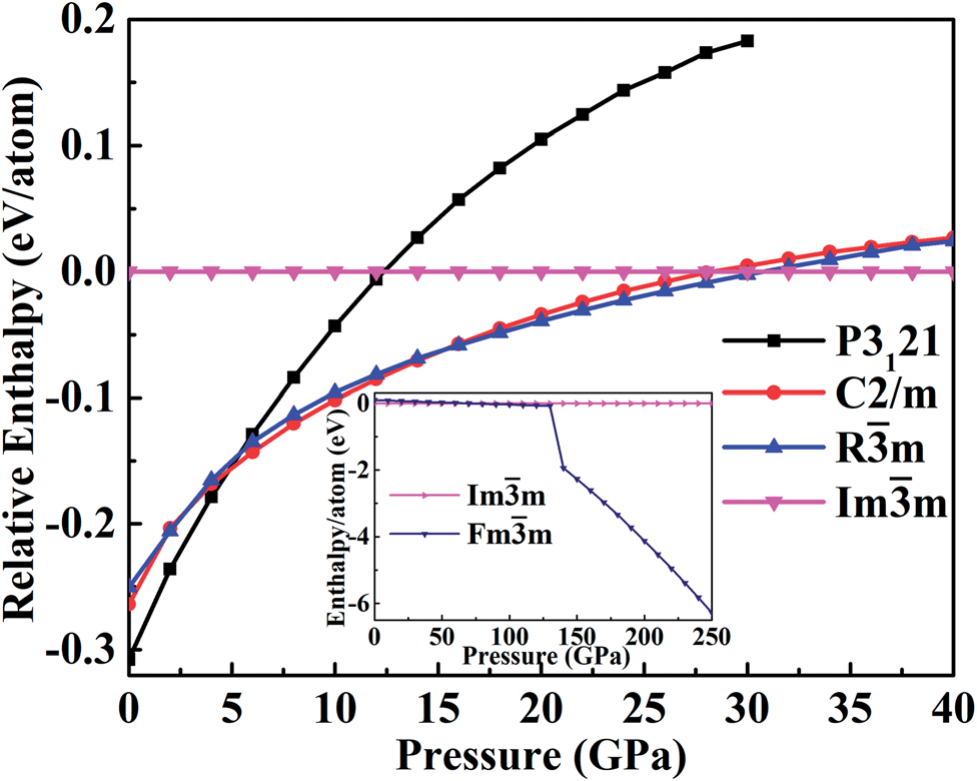
The calculated enthalpies relative to the high-pressure *Im*3̄*m* phase as a function of the pressure.

The enthalpy as a function of the pressure, reported in Fig. 2, can help to identify the phase transitions. This confirms that the *P*3_1_21 phase is the most stable structure up to 4 GPa. As the pressure increases up to 5 GPa, the *C*2/*m* phase has lower enthalpy than that of *P*3_1_21 phase and becomes stable at 6 GPa. At pressure less than 15 GPa, the monoclinic *C*2/*m* phase transforms into the trigonal *R*3̄*m* phase. Experimentally, this transition was suggested in the pressure range of 10.6–27 GPa.^18,20^ Furthermore, the transformation from the trigonal *R*3̄*m* to the cubic *Im*3̄*m* occurs at about 30 GPa. The X-ray-diffraction experiments^20^ report the transition from the rhombohedral structure to the body-centered-cubic structure at 27 ± 3 GPa. The cubic *Im*3̄*m* structure then transform into the fcc *Fm*3̄*m* structure at pressure larger than 100 GPa.

**Fig. 3:**
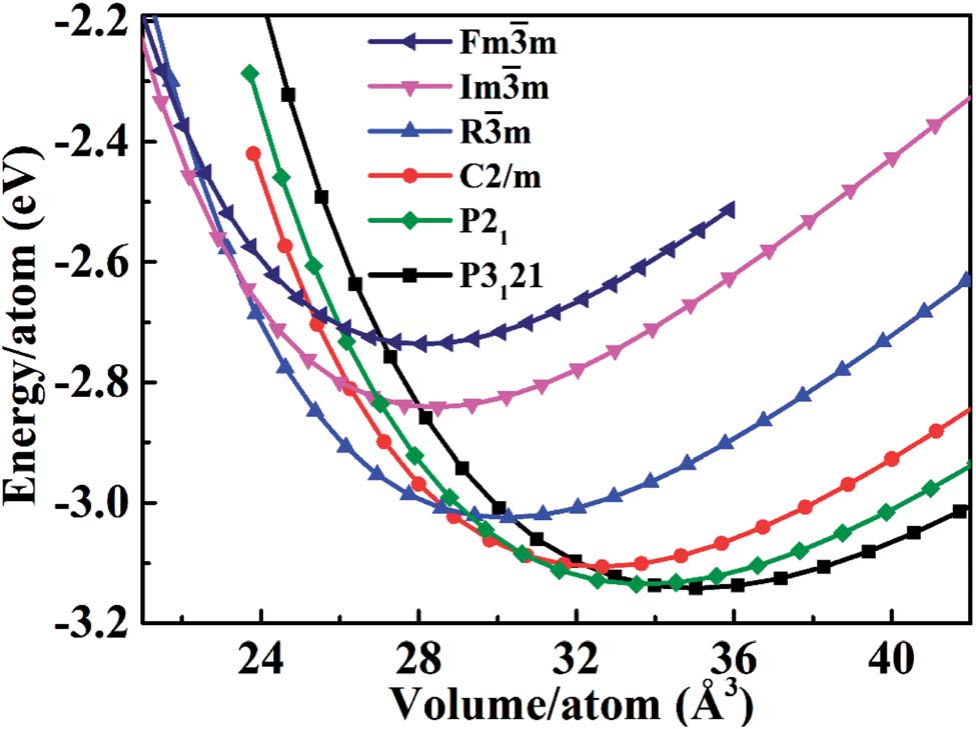
The total energy as a function of the volume (at *P* = 0).

We calculated the bulk modulus and its first derivative with respect the volume by fitting, for each phase, the total energy as a function of the volume with a third-order Birch–Murnaghan equation:^39^1
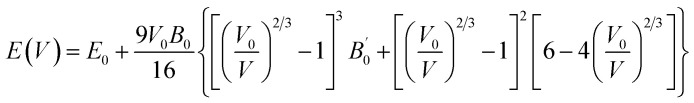
where *V*_0_ is the volume per formula unit at ambient pressure, *V* is the volume per formula unit at pressure *P* given in GPa, *B*_0_ is the isothermal bulk modulus, and 
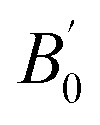
 is the first derivative of the bulk modulus with respect to the pressure. The values of *B*_0_ and 
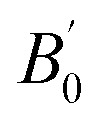
 are listed in Table 1. Our energy-volume curves, shown in Fig. 3, are in good agreement with computational works reported in the literature.^40^ From the energy-volume curves we can confirm that the monoclinic structure *C*2/*m* represents a phase stable at intermediate pressure between the ground-state, trigonal *P*3_1_21, and the high pressure trigonal *R*3̄*m* structure.

Actually, the structural complexity of tellurium under pressure remains elusive even experimentally.^20,21,23^ The very early X-ray diffraction study reported a monoclinic to orthorhombic phase transition at 6.6 GPa.^16^ However, such orthorhombic state might be attributed to a different representation of the monoclinic *P*2_1_ structure, which we found having the lattice angle *β* very close to 90°, in good agreement with the experimentally reported phase transitions.^29^ Our enthalpy-pressure curves show that the transition sequence is: trigonal *P*3_1_21 to monoclinic *C*2/*m* (at 6 GPa) to trigonal *R*3̄*m* (at 10 GPa) to cubic *Im*3̄*m* (at 28 GPa) and then to the cubic *Fm*3̄*m* at pressure as large as 200 GPa.

**Fig. 4:**
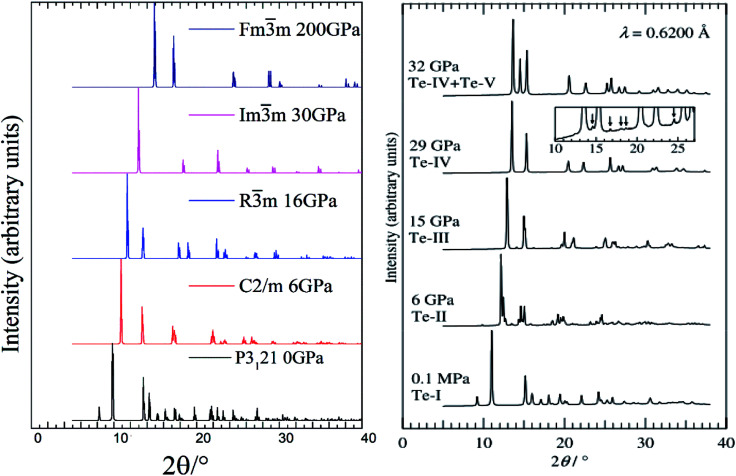
Simulated XRD patterns of the optimized structures at different pressures (left panel) compared with experiment work (right panel).^20^

Our calculated XRD patterns of the *P*3_1_21, *C*2/*m*, *R*3̄*m*, *Im*3̄*m* and *Fm*3̄*m* structures are shown in Fig. 4 for a comparison with experiments.^20^ We have found excellent consistency between the calculated and the experimental XRD patterns of *P*3_1_21 phase.^20^ As the pressure increases, the small peak below 10° completely disappears in the *C*2/*m* and *R*3̄*m* phases. Very interestingly, if we combine our simulated XRD of *R*3̄*m* and *Im*3̄*m* structures at 28 GPa, we could perfectly reproduce the phase coexistence, Te-IV + Te-V, reported at 32 GPa experimentally.^20^

**Fig. 5:**
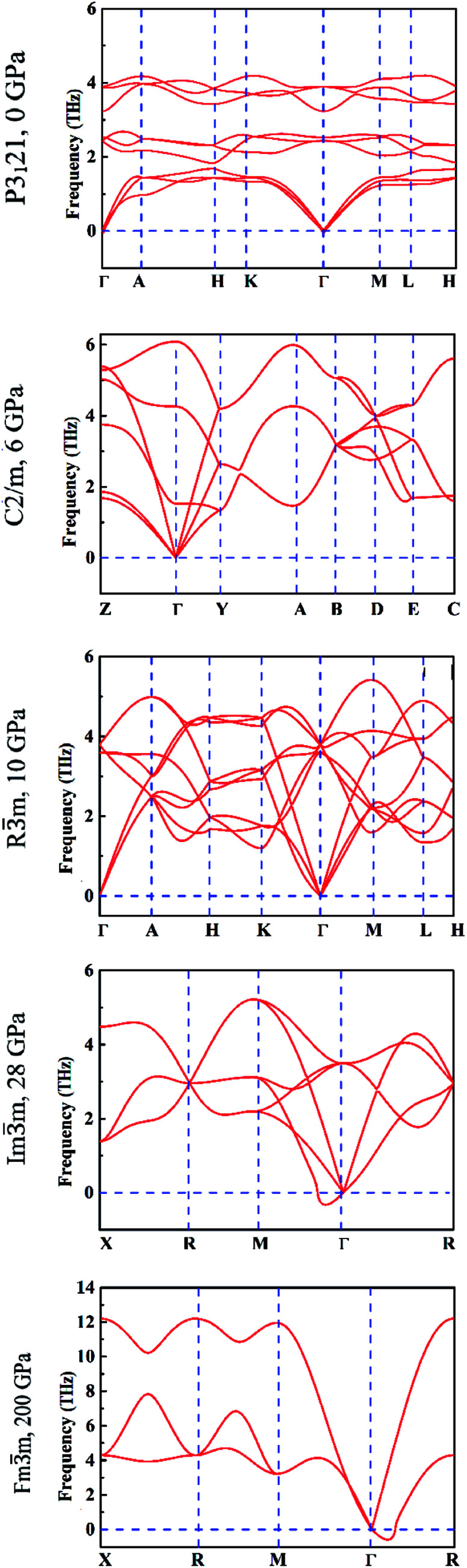
The calculated phonon dispersion curves under different hydrostatic pressures.

It is well known that the soft phonon modes with negative frequencies are indicative of the dynamical instability of a crystal. To get a better understanding of the dynamical and mechanical stability of these phases, we calculated the phonon dispersions and elastic constants based on the first principles and direct force-constant approaches by using the Phonopy package.^31^ The calculated phonon spectra of different tellurium phases are displayed in Fig. 5. We observe that the interplay between the strong covalent intra-chain and weak inter-chain interactions in trigonal *P*3_1_21 tellurium^41,42^ gives rise to the phonon band gap between the lower and higher optical phonon branches. This is consistent with the DFT calculation results^6^ and also the experimental dispersion curves reported in the literature.^43^

The absence of negative phonon frequencies or any soft phonon mode, confirms the lattice stability of the five structures. In particular, the monoclinic *C*2/*m* structure is stable at 6 GPa, the trigonal *R*3̄*m* structure at 10 GPa. The two cubic structures are stable at pressure larger than 28 GPa and 200 GPa, respectively, at which the corresponding transitions occur. The dynamical instabilities of the two cubic structures visible in the corresponding phonon dispersion curves in Fig. 5 are attributable to translational modes at the respective phase transitions.

In addition, we calculated the phonon spectra of the monoclinic *P*2_1_ phase at 2 GPa and found that it is similar to the trigonal *P*3_1_21 structure, confirming that it is not a distinct phase.

**Fig. 6:**
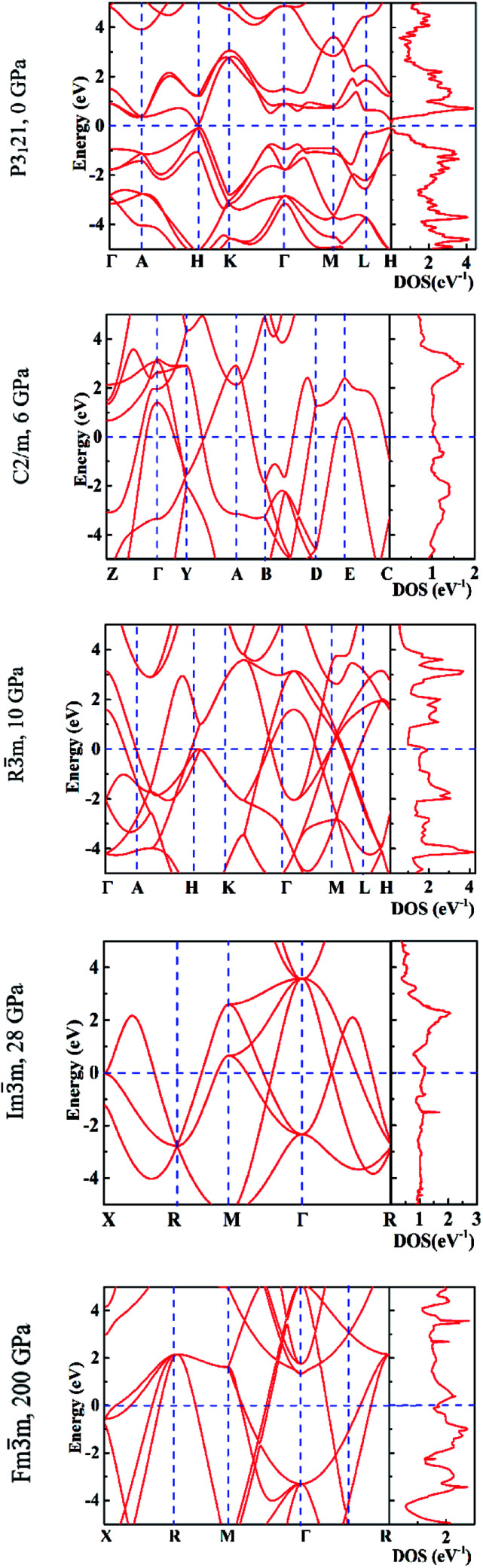
The calculated electronic band structures (left panels) and density of states (right panels).

In Fig. 6 we report the calculated electronic band structures and the electronic density of states (DOS) for *P*3_1_21 at 0 GPa, *C*2/*m* at 6 GPa, *R*3̄*m* at 10 GPa, *Im*3̄*m* at 28 GPa and *Fm*3̄*m* at 200 GPa. We found that the *P*3_1_21 phase has a direct band gap of 0.15 eV by using PBE functional and 0.55 eV by using a hybrid functional. This is in good agreement with the previous *ab initio* calculations,^40^ and the experimental value of a direct gap of 0.33 eV.^44^ The pressure induces the phase transition from semiconductor to metal, as all phases at pressure above zero are metallic.^16,18,38^

## Conclusions

We investigated the allotropes of tellurium crystal under pressure by combining first-principles total energy calculations (VASP) and crystal structure prediction methodology (CALYPSO). The calculated enthalpy–volume curves indicate that tellurium transforms from the ground state, trigonal *P*3_1_21 phase, to the α-Po type structure, trigonal space group *R*3̄*m*, *via* the monoclinic *C*2/*m* phase in the range of 0–10 GPa. As increasing the pressure, the rhombohedral structure of the *R*3̄*m* phase transforms into a stable body centered cubic structure (bcc), space group *Im*3̄*m*, at 28 GPa. At higher pressure, larger than 100 GPa, the bcc structure transforms into a face centered cubic structure, space group *Fm*3̄*m*, whose lattice turns stable at 200 GPa. The comparison of the calculated and experimental XRD patterns indicates a good agreement of the sequence of the phases upon the applied pressure.^20^ We calculated the phonon spectra of all phases and found that the monoclinic *C*2/*m* is stable at 6 GPa, the trigonal *R*3̄*m* at 10 GPa, the cubic *Im*3̄*m* at 28 GPa and the cubic *Fm*3̄*m* at 200 GPa. In addition, the band structures confirmed the pressure-induced transition from the ground state semiconductor phase to the metallic phases at pressure larger than 2 GPa.

## Conflicts of interest

There are no conflicts to declare.

## Supplementary Material
